# Association Between Concerns About COVID-19 Infection and Blood Donation Intention: Cross-Sectional Survey Study Through a Mobile Communication Platform

**DOI:** 10.2196/46588

**Published:** 2023-11-09

**Authors:** Qiuyue Hu, Wei Hu, Lingling Pan, Wenjuan Han, Yue Zheng

**Affiliations:** 1 Blood Center of Zhejiang Province Hangzhou China; 2 Key Laboratory of Blood Safety Research, Zhejiang Province Hangzhou China

**Keywords:** COVID-19, blood donation, worry, concern, intention, blood, blood transfusion, cognition, blood donor, communication, questionnaire, behavior control

## Abstract

**Background:**

The ongoing COVID-19 pandemic has had an unprecedented impact on blood transfusion and collection. At the beginning of the pandemic, most blood transfusion services had a tough challenge in maintaining an optimal blood inventory.

**Objective:**

This study aims to understand the public's psychological cognition and intention toward blood donation as well as the factors influencing their worries. We aimed to find a solution for increasing blood donations and provide a scientific reference for policy formulation regarding blood donation during the COVID-19 pandemic and in the future.

**Methods:**

A random survey with a 14-item scale on worries related to blood donation was conducted from December 31, 2022, to January 3, 2023, among residents aged 18-60 years in Zhejiang province via SMS text messaging. The results of 8 worry items in this study were compared with the survey results of March 2022, during which COVID-19 was not considered as an epidemic in Zhejiang province. Chi-square test and logistic regression analysis were performed to analyze the factors affecting respondents’ blood donation intention and concerns. The degree of worry about blood donation was assigned from 1 (completely disagree) to 5 (completely agree), and 2-sided *t* tests were performed to analyze the differences in blood donation intention and worries about blood donation.

**Results:**

In total, 1254 valid questionnaire responses were obtained. Males accounted for 62.36% (782/1254) of the sample, 78.39% (983/1254) were 18-45 years old, 60.61% (760/1254) had a university education, and 69.06% (866/1254) had no previous blood donation experience. Approximately 36.52% (458/1254) of the public clearly expressed that they had blood donation worries regarding COVID-19. The main concerns of the respondents were temporary physical weakness caused by blood donation, their own physical conditions not meeting the requirements of blood donation, inconvenient location and working hours for blood donation, and family (or friends) worrying about blood donation. Compared with the results in 2022, the results in 2023 regarding the harmful effects of blood donation on health, temporary physical weakness, infection in donated blood, and family (friends) worrying increased significantly (*P*<.001). The factors influencing blood donation worries regarding COVID-19 were COVID-19 infection status, adverse reactions to the donated blood, family (or friends) worrying, and unsatisfactory blood donation experience. The factors influencing blood donation intention were gender, age, previous blood donation times, blood donation worries regarding COVID-19, harmful effects of blood donation on health, and blood donation anxiety.

**Conclusions:**

Blood transfusion services should make full use of the recovery phase of COVID-19 infection as an important time point, publicize the blood donation process and operation standardization, reduce the public's concerns about blood donation, correct negative evaluations, and increase perceived behavioral control and subjective norms.

## Introduction

The ongoing COVID-19 pandemic has had an unprecedented impact on blood transfusion and collection services [1]. In particular, at the beginning of the COVID-19 pandemic in 2020, most blood transfusion services (BTSs) faced major and universal challenges to maintain an optimal blood inventory for clinical treatment needs [2-8]. On December 7, 2022, the Comprehensive Group of the State Council of the People’s Republic of China’s Joint Prevention and Control Mechanism against the COVID-19 epidemic issued 10 new measures to further optimize the response to COVID-19, thereby making more efficient use of prevention and control resources and better coordination of epidemic prevention and control with economic and social development [9]. They issued the overall plan “secondary classification, secondary management” to be implemented for COVID-19 on December 26, 2022. The plan was that from January 8, 2023, China will adjust its policy on COVID-19 infection from “secondary classification, primary management” to “secondary classification, secondary management” [10]. According to the Law of the People’s Republic of China on the Prevention and Control of Infectious Diseases [11], COVID-19–infected persons will no longer be isolated, judged as close contacts, or designated as living in high- or low-risk areas, etc. Since December 10, 2022, waves of COVID-19 hit various provinces in China one after another. In Italy, Brazil, Spain, Greece, Iran, and many other countries, blood donations reduced since the start of the COVID-19 pandemic [12-15]. Approximately 77.4% (range 10%-50%) of the BTSs in 26 low- to middle-income countries reported a drop in blood donations [16]. The decrease in blood donations was mostly due to lockdown measures, reduced number of mobile blood donation stations, fear of contracting diseases, quarantine enforcement, and ineligibility to donate blood [1,14]. A study in Saudi Arabia showed that the biggest obstacle to blood donations during the COVID-19 pandemic was the lack of fit health [17]. Meanwhile, a study [18] has shown that reduction in admissions due to COVID-19 pandemic resulted in only a moderate decrease in the need for blood transfusion. The cancellation of elective surgery during the pandemic resulted in 14% reduction in blood transfusion. Blood transfusion is necessary for urgent surgery, acute bleeding, or as supportive treatment for chemotherapy-induced aplasia [18]. BTSs have been facing a severe challenge of a sharp reduction in the number of qualified blood donors and the amount of blood donated and have been under unprecedented pressure since the beginning of the COVID-19 pandemic.

This study was conducted in 2023, during which national policies in China had eliminated the quarantine measures for COVID-19. Therefore, there was no quarantine or isolation of sick or infected persons or cancellation of elective surgery. All types of surgeries, including elective surgery, continued as usual; therefore, the need for blood transfusion had not decreased. Because of the decrease in the number of blood donors and the increase in demand for blood supply after the COVID-19 pandemic, this gap between blood supply and demand needs to be urgently addressed. It is necessary to study people's views, cognition, and intentions regarding voluntary nonremunerated blood donation after the removal of lockdown measures, physical isolation, and other external influencing factors. It is important to explore the reasons underlying the sharp decline in the number of blood donors and to determine the measures that BTSs need to take to address these obstacles as soon as possible. Many studies [3,4,19] have focused on the challenges of maintaining blood supply in countries with mixed blood donation models and their responses to blood supply, but they have not adequately focused on the challenges faced. However, a study in Hong Kong has focused on the institutional, social, and perceived factors influencing current blood donors as well as provided a macro understanding of the blood donation behavior [13]. A study in Australia assessed blood donor status by using the integrated protection motivation theory, organizational trust, and theory of planned behavior framework. They found that self-efficacy and approval from others, underpinned by coping appraisals and organizational trust, play a critical role in the intention to donate [20]. A study in Jiangsu, China, used machine learning methods to predict blood donation intentions and to improve the recruitment rate of blood donors [21].

Does donating blood lead to contracting COVID-19? Although some studies have shown that fear of infection was the reason for people's reluctance to donate blood during the COVID-19 pandemic [5,6], a Syrian study showed that stress, anger, and fear of infection were not associated with blood donation and its patterns [22]. Therefore, there is no strong evidence regarding the concerns people have about donating blood during the pandemic, the risks and threats perceived by the people, and whether these affect people's willingness to donate blood.

Zhejiang is located in eastern China and has a total population of 50,957,800 persons [23]. There are 38 BTSs in Zhejiang. For the first time, under the guidance of framework of the extended theory of planned behavior, we developed the Blood Donation Worry Scale during the COVID-19 pandemic. The population in Zhejiang was considered to understand the public's awareness and worries about blood donation in the absence of lockdown measures during the COVID-19 pandemic, to explore the factors that lead to blood donation worries and that influence blood donation intentions, and to provide suggestions to improve the blood donation supply according to these influencing factors. Our study is expected to provide a scientific reference for policy formulation regarding voluntary nonremunerated blood donation during the COVID-19 pandemic and in the future.

## Methods

### Research Design

The eligible age for blood donation in China is 18-55 years. However, if experienced blood donors who have had no negative reaction in the past and meet the health requirements volunteer to donate blood again, the age limit can be extended to 60 years [24]. In this cross-sectional study, we developed a scale for people aged 18-60 years, residing in 11 cities in the Zhejiang province. A short message containing a link to the scale was sent to the residents by using a mobile communication platform, and a web-based survey was conducted to collect the residents’ views, cognition, worries, and intention to donate blood in the context of the COVID-19 pandemic.

### Sample Size Calculation

According to research on residents' anxiety during the COVID-19 period in Zhejiang province, the positive rate of anxiety among residents was 41.41% [25]. The confidence level of the sample size was 95%, while the set α was .05, z_α/2_ was 1.96, and the tolerance error δ was 0.04, based on the sample size calculation formula: n=z^2^_α/2_ *P* (1- p) / δ^2^. The required sample size was calculated to be 583 individuals. Assuming that 20% of the responses would be problematic, the sample size was adjusted to 729 for this study.

### Scale Development

Based on interviews with blood donors and a review of relevant literature, we developed the Blood Donation Worry Scale by using the Likert scale method, drawing from the Blood Donation Anxiety Scale designed based on the extended theory of planned behavior [26,27] and the Negative Outcome Expectation Scale [28] in the context of the COVID-19 pandemic. Using the Delphi method, experts in the fields of blood donation, epidemiology, social medicine, and other relevant fields were invited to perform 3 validation modifications, and 10 pilot tests were performed. The scale was finalized after fine-tuning the statements. The scale was divided into 2 parts: the first part was the survey description and informed consent form and the second part was the content of the scale. The content of the scale consisted of basic information and subscales. Basic demographic factors such as sex, age, education level, previous blood donation times, and current COVID-19 infection status were included. The current COVID-19 infection status was divided into 4 categories: uninfected, positive infection, recovered within 7 days since infection, and recovered after more than 7 days since infection. According to the Guidelines for the Prevention and Control of COVID-19 infection in BTSs (edition II) issued by China [29], individuals infected with COVID-19, except for severe and critical cases, can donate blood at 7 days after the last positive result. The subscale had 3 parts: intention to donate blood, blood donation worries regarding COVID-19, and the specific content of blood donation worries regarding COVID-19 summed up in 14 items. The item of “intention to donate blood” was divided into 7 categories: (1) I have never heard of voluntary blood donation; (2) I have heard of voluntary blood donation, but I have not considering blood donation; (3) I am still struggling to donate blood; (4) I have decided not to donate blood; (5) I have decided to donate blood but have not taken any action yet; (6) I have decided to donate blood, but I am currently unable to donate blood due to the requirements of blood donation (such as the interval time after infection); and (7) I have donated blood recently. For convenience in the analysis, the former 4 categories were grouped as “no intention to donate blood or not yet,” and the latter 3 categories were grouped as “intention to donate blood.”

The item of “have you had any worries (hereinafter referred to as blood donation worries regarding COVID-19) about your ability to donate blood after being infected with COVID-19 (or assumed infected)?” Specifically, this refers to the psychological state of refusing to donate blood even if the policy and physical conditions permit to do so (Textbox 1). It also includes the behavior of participating in blood donation but with doubts. In particular, this item assessed the respondents' thoughts or behaviors about their ability to donate blood in the context of the COVID-19 pandemic. Based on the response to this item, respondents were classified as part of the worried group if they answered yes and the non–worried group if they answered no. Further, to understand the specific concerns of these 2 groups about blood donation worries regarding COVID-19, we listed 14 common worries and used a 5-point Likert subscale to assess their specific worries. According to the literature and previous research experience [1,15,16], this 14-item scale was guided by the extended theory of planned behavior and based on the attitude, perceived behavior control, and subjective norms of the theory of planned behavior while also extending the factors of blood donation anxiety and negative outcome expectation. Specifically, item a corresponds to the attitude factor; item l and item i correspond to the perceived behavior control factor; item f, item g, and item h correspond to the subjective norm factor; item n represents the blood donation anxiety factor; and items b, c, d, e, j, k, and m correspond to the negative outcome expectation factor. Reliability and validity analyses were performed on the worry scale with 14 items. The results showed that Cronbach α was .911 and Guttman split-half was 0.879, both of which were above .70, indicating good internal consistency reliability of the scale. The Kaiser-Meyer-Olkin Measure of Sampling Adequacy was 0.927, which was greater than 0.800, indicating a strong correlation between variables and meeting the requirements.

**The scale of blood donation worries in the context of the COVID-19 pandemic.**

**Blood donation worry scale**

Have you had any worries (hereinafter referred to as blood donation worries regarding COVID-19) about your ability to donate blood after being infected with COVID-19 (or assumed infected)? Specifically, this refers to the psychological state of refusing to donate blood, even if the policy and physical conditions permit it. It also includes the behavior of participating in blood donation but still having doubts.

The results of this study were compared with the concerns of the population in Zhejiang province collected in a cross-sectional study (Q Hu, unpublished data, March 2022), which was performed when COVID-19 was not considered as an epidemic in Zhejiang owing to the control measures implemented at that time. That study also had 8 items with the same or similar content, as shown in Table 1.

- Yes/No

According to your actual situation, please choose the option that best fits your idea in the following sentences. A. completely disagree B. disagree C. neutral/don’t know D. agree E. completely agree

- Item a: Blood donation is harmful to health.
- Item b: Blood donation will cause temporary weakness.
- Item c: I am worried about adverse reactions to blood donation.
- Item d: Donated blood may be infected with diseases.
- Item e: Blood donation will increase the risk of COVID-19 infection or secondary infection.
- Item f: Family or friends would be worried if I participated in blood donation.
- Item g: Family or friends would be unhappy or angry if I participated in blood donation.
- Item h: My family would criticize me for donating blood.
- Item i: The blood donation site’s location and working hours were inconvenient for me.
- Item j: Blood donation was voluntary and nonremunerated, but the blood was remunerated. I am worried that the blood I donate will be taken for profit.
- Item k: I am afraid of needles used for blood donation.
- Item l: I am worried that my physical condition does not meet the blood donation requirements.
- Item m: Dissatisfaction with the blood donation experience (whether when I donated blood or what I heard from others) caused me to worry about donating blood.
- Item n: If I am asked to donate blood or donate blood again, I will feel distressed and anxious.

**Table 1:** Comparison of the scales used in this study and in the study conducted during the non–COVID-19 pandemic stage (Q Hu, unpublished data, March 2022).

Item from Textbox 1	Items of this study	Items of the study during non–COVID-19 pandemic stage
a	Blood donation is harmful to health.	Blood donation will not damage the body.
b	Blood donation will cause temporary weakness.	How scared or worried are you about appearing weak during or after blood donation?
c	I am worried about the adverse reactions to blood donation.	Have you ever experienced dizziness, weakness, or a mild headache during or after donating blood?
d	Donated blood may be infected with diseases.	Blood donated will not be infected with diseases.
f	Family or friends would be worried if I participated in blood donation.	Most of the people who are important to me think I should donate blood or donate again.
g	Family or friends would be unhappy or angry if I participated in blood donation.	Most of the people who are important to me will support and encourage me to donate again.
m	Dissatisfaction with the blood donation experience (whether when I donated blood or what I heard from others) caused me to worry about donating blood.	Dissatisfaction with the blood donation experience (whether when I have donated blood or heard from others) causes me to worry about donating blood.
n	If I am asked to donate blood or donate blood again, I will feel distressed and anxious.	If I am asked to donate blood or donate blood again, I will feel distressed and anxious.

Items a, d, f, and g in both the studies reflected the same essence, but the expression was in the opposite direction in the study during the non–COVID-19 stage. These 4 items were subjected to comparative analysis after corresponding processing by the scoring method. Items b and c were similar in content in both the studies, with the same direction in the expression, and items m and n were exactly the same, facilitating comparative analysis.

### Ethics Approval

This study was approved by the regional ethics committee of the Blood Center of Zhejiang Province (approval 2023-021). Each respondent carefully read and filled the informed consent form, which evaluated both the respondent's understanding ability and the screening of research participants. Respondents indicated their understanding of the study and gave their authorization by ticking the agreement box. The informed consent form included the following content: I understand that I can withdraw from this investigation at any time. I understand that the information I provided while participating in this survey will be kept confidential. I understand the purpose and nature of this investigation. I agree that the researcher can use the data collected from this survey for analysis and research. I agree to participate in this investigation.

### Data Collection

Using the platform of the Zhejiang Mobile Communication Company, we conducted a stratified random sampling survey among the public according to age. From December 31, 2022, to January 3, 2023, a total of 100,151 invitation text messages were sent to people aged 18-60 years in the Zhejiang province. The text messages contained links to the scales. Each person who successfully completed the scale was rewarded with ¥5 (¥1=US $0.7). On January 4, a total of 1254 scales were collected, with a recovery rate of 1.25%, and all these scales were validated, with an effective rate of 100%, which met the sample size requirements.

### Statistical Methods

SPSS (version 21.0; IBM Corp) was used to organize and analyze the data collected in this study, and the count data were expressed as n (%). Chi-square test and logistic regression analysis were performed to analyze the factors affecting the intentions and worries of the respondents’ regarding blood donation. The degree of worry about blood donation was assigned from 1 point (completely disagree) to 5 points (completely agree), represented by mean (SEM), and 2-sided *t* tests were performed to analyze the difference in blood donation intention and blood donation worries. Statistical significance was set at *P*<.05. In the logistic regression analysis, blood donation worries regarding COVID-19 and blood donation intention were defined as dependent variables. The independent variables were assigned as follows: gender (female=0, male=1), age (18-25 years=1, 26-35 years=2, 36-45 years=3, 46-55 years=4, 56-60 years=5), education (high school or lower than high school=1, junior college=2, university=3, postgraduate=4, other=5), current COVID-19 infection status (uninfected=1, recovered after more than 7 days since infection=2, recovered within less than 7 days since infection=3, positive infection=4), number of previous blood donations (0 time=1, 1 time=2, 2 times=3, ≥3 times=4), blood donation worries regarding COVID-19 (no=0, yes=1), and intention to donate blood (no=0, yes=1). The worry items were assigned using the Likert scale method; the degree of worry was assigned from 1 point (completely disagree) to 5 points (completely agree). We took factors that correspond to assigning the minimum value as a reference category in logistic regression analysis.

## Results

### Basic Information of the Participants

As shown in Table 2, males accounted for 62.36% (782/1254) of the total respondents. Most of them were 18-25 years old (410/1254, 32.70%), followed by 36-45 years of age (364/1254, 29.03%), and most of them had a college education (760/1254, 60.61%). The number of previous blood donations was 0 time for 69.06% (866/1254) of the respondents; the respondents were mainly novel blood donors or non–blood donors. The survey showed that only 22.25% (279/1254) of the respondents were not infected with COVID-19, while 77.75% (975/1254) of the respondents were infected or in the postinfection stage, indicating that the infection rate was already high at that time. Of the participants, 36.52% (458/1254) answered yes to the item of blood donation worries regarding COVID-19, which was the first item in Textbox 1. Thus, these participants worried about whether they could donate blood after being infected with COVID-19 (or assuming that they were infected) in their self-assessment. According to this answer, the respondents were divided into worried group or non–worried group, and the 2 groups were compared via chi-square test. Table 2 shows that there was a significant difference (*P*<.001) between the status of COVID-19 infection and whether they had blood donation worries regarding COVID-19. The proportion of blood donation worries regarding COVID-19 was the highest (76/155, 49%) among respondents in the COVID-19 infection period, and this proportion was significantly higher than that in other periods (*P*<.001).

**Table 2:** Basic demographic information of the respondents and a comparative analysis of the blood donation behavior of the worried group and the non–worried group.

			Total (N=1254), n (%)		Worried group (n=458), n (%)		Non–worried group (n=796), n (%)		Chi-square *(df)*			*P* value	
**Gender**										0.03 (2)			.87
	Male	782 (62.4)		287 (36.7)		495 (63.3)							
	Female	472 (37.6)		171 (36.2)		301 (63.8)							
**Age (years)**										7.1 (8)			.13
	18-25	410 (32.7)		143 (34.9)		267 (65.1)							
	26-35	209 (16.7)		87 (41.6)		122 (58.4)							
	36-45	364 (29)		142 (39)		222 (61)							
	46-55	261 (20.8)		84 (32.2)		177 (67.8）							
	56-60	10 (0.8)		2 (20)		8 (80)							
**Education**										1.0 (8)			.91
	≤High school	406 (32.4)		145 (35.7)		261 (64.3）							
	University	760 (60.6)		279 (36.7)		481 (63.3)							
	Postgraduate	50 (3.9)		19 (38)		31 (62)							
	Other	38 (3)		15 (39.5)		23 (60.5）							
**Number of previous blood donations**										6.4 (6)			.09
	0 time	866 (69)		324 (37.4)		542 (62.6）							
	1 time	154 (12.3)		64 (41.6)		90 (58.4）							
	2 times	66 (5.3)		19 (28.8)		47 (71.2）							
	≥3 times	168 (13.4)		51 (30.4)		117 (69.6）							
**COVID-19 status**										101.6 (6)			<.001
	Uninfected	279 (22.25)		83 (29.7)		196 (70.3)							
	Recovered after more than 7 days since infection	461 (36.76)		168 (36.4)		293 (63.6)							
	Recovered within less than 7 days since infection	359 (28.63)		38 (10.6)		321 (89.4)							
	Positive infection	155 (12.36)		76 (49)		79 (51)							

### Analysis of the Worry-Related Items

Our study identified 14 specific worry items and found that the 4 most worrisome aspects for people were that blood donation will cause temporary weakness (mean 3.21, SEM 1.177), physical condition does not meet the blood donation requirements (mean 2.90, SEM 1.180), the location and working hours of blood donation are not convenient (mean 2.83, SEM 1.046), and my family (friends) will be worried about my participation in blood donation (mean 2.68, SEM 1.118) (Table 3).

**Table 3:** Results of the worries about blood donation in the context of the COVID-19 pandemic in the web-based survey analysis.

Item from Textbox 1	Description	Mean (SEM)
a	Blood donation is harmful for health.	2.12 (0.969)
h	Family would criticize me for donating blood.	2.19 (0.971)
g	Family or friends would be unhappy or angry if I participated in blood donation.	2.29 (0.977)
n	If I am asked to donate blood or donate blood again, I will feel distressed and anxious.	2.33 (1.010)
e	Blood donation will increase the risk of COVID-19 infection or secondary infection.	2.37 (0.952)
d	Donated blood may be infected with diseases.	2.39 (1.042)
m	Dissatisfaction with the blood donation experience (whether when I have donated blood or heard from others) causes me to worry about donating blood.	2.43 (1.054)
k	I am afraid of the needles used for blood donation.	2.48 (1.142)
c	I am worried about the adverse reactions after blood donation.	2.59 (1.084)
j	Blood donation was voluntary and nonremunerated, but using blood was remunerated. I am worried that the blood I donated will be used for profit.	2.62 (1.138)
f	Family or friends would be worried if I participated in blood donation.	2.68 (1.118)
i	The blood donation site’s location and working hours were inconvenient for me.	2.83 (1.046)
l	I am worried that my physical condition does not meet the blood donation requirements.	2.90 (1.180)
b	Blood donation will cause temporary weakness.	3.21 (1.177)

### Comparison Between the Specific Worry Items During the COVID-19 Pandemic and During the Noninfectious Period in 2022

In this study, the 14 worry items of the respondents were compared with the worry items collected in a previous cross-sectional study conducted by Q Hu (unpublished data, March 2022), which collected data from 1198 participants in Zhejiang to determine people’s basic psychology toward blood donation. A comparison of 8 items (Multimedia Appendix 1) showed that all items were significantly different except for “family (friends) will be unhappy (angry) if I participated in blood donation.” Among them, the extent of worries about the items on blood donation is harmful to health, blood donation will cause temporary weakness, donated blood may be infected with diseases, and family or friends would be worried if I participated in blood donation was significantly higher (all *P*<.001) than that in March 2022. However, the worries about adverse reactions to blood donation; dissatisfaction with the blood donation experience (whether when I donated blood or what I heard from others, causes me to worry about donating blood); and if I am asked to donate blood or donate blood again, I will feel distressed and anxious were lower than those in March 2022 during the non–COVID-19 pandemic.

### Logistic Regression Analysis of Blood Donation Worries Regarding COVID-19

A logistic regression analysis was conducted on the blood donation worries regarding COVID-19 as the dependent variable. As shown in Table 4, the items COVID-19 infection status, worry about adverse reactions to blood donation, family or friends worrying with regard to blood donation, and dissatisfaction with the blood donation experience were the factors influencing blood donation worries regarding COVID-19.

**Table 4:** Logistic regression analysis of blood donation worries regarding COVID-19.

	β	SE	Chi-square *(df)*	*P* value	Odds ratio (95% CI)
COVID-19 status	.219	0.064	11.8 (1)	.001	1.245 (1.098-1.410)
I am worried about the adverse reactions to blood donation	.224	0.071	10.1 (1)	.002	1.252 (1.090-1.438)
Family or friends would be unhappy or angry if I participated in blood donation	.171	0.065	6.9 (1)	.008	1.186 (1.045-1.347)
Dissatisfaction with the blood donation experience (whether when I donated blood or what I heard from others) causes me to worry about donating blood	.209	0.070	8.8 (1)	.003	1.232 (1.073-1.414)
Constant	–2.645	0.254	108.5 (1)	<.001	0.071

### Logistic Regression Analysis of Blood Donation Intention During the COVID-19 Pandemic

Table 5 shows that the 7 influencing factors of blood donation intention were gender, age, number of previous blood donations, blood donation worries regarding COVID-19, harmful effects of blood donation on health, inability of the physical condition to meet the blood donation requirements, and feelings of distress and anxiety when donating blood or donating blood again. Specifically, males, older people, more previous blood donations, no blood donation worries regarding COVID-19, less worry about blood donation being harmful to health, and less anxiety about blood donation were associated with stronger intentions to donate blood.

**Table 5:** Logistic regression analysis of the influencing factors of blood donation intention.

	β	SE	Chi-square *(df)*	*P* value	Odds ratio (95% CI)
Gender	–.368	0.148	6.2 (1)	.01	0.692 (0.518-0.925)
Age	.301	0.063	23.2 (1)	<.001	1.352 (1.196-1.528)
Number of previous blood donations	1.599	0.189	71.7 (1)	<.001	4.948 (3.417-7.164)
Blood donation worries regarding COVID-19	–.289	0.145	4.1 (1)	.045	0.749 (0.564-0.994)
Blood donation is harmful to health	–.397	0.082	23.4 (1)	<.001	0.672 (0.573-0.790)
If I am asked to donate blood or donate blood again, I will feel distressed and anxious.	–0.679	0.087	61.5 (1)	<.001	0.507 (0.428-0.601)
Constant	1.828	0.314	33.7 (1)	<.001	6.222

## Discussion

### Summary

Guided by the framework of the extended theory of planned behavior, this study shows that the public had a certain degree of worry about blood donation during the COVID-19 pandemic, mainly in the following 4 aspects: blood donation will cause temporary weakness, I am worried that my physical condition does not meet the blood donation requirements, the blood donation site’s location and working hours were inconvenient for me, and family or friends would be worried if I participated in blood donation. Blood donation worries regarding COVID-19 were influenced by the status of COVID-19 infection, worries about adverse reactions to blood donation, worries of family or friends regarding blood donation, and dissatisfaction with the blood donation experience. The main factors affecting the willingness to donate blood were the blood donation worries regarding COVID-19, along with gender, age, number of previous blood donations, perception that donating blood is harmful to the body, and the anxiety of donating blood.

The data collection period for this survey was from December 31, 2022, to January 3, 2023. The survey showed that the infection rate during that time reached 77.75% (975/1254), which was slightly lower than the infection rate of 89% in Henan province as of January 6, 2023 [30]. During the period when COVID-19 was prevalent in Zhejiang province, blood collection and supply were very challenging. The collected data reflected psychological factors such as people's cognition, worries, and intention to donate blood during the COVID-19 pandemic. At this stage, 36.52% (458/1254) of the respondents clearly expressed blood donation worries relating to COVID-19. This indicates that although the public had certain concerns, these sentiments were not dominant. However, is the impact of the COVID-19 pandemic on blood donation limited to these factors?

### Four Main Worries of the Public Regarding Blood Donation

The 4 main worries of the public regarding blood donation during the COVID-19 pandemic reflected a decline in the perceived behavioral control and subjective norms toward blood donation as well as the negative impact of attitudes toward blood donation.

The first worry of the public regarding blood donation during the COVID-19 pandemic was the effect of the COVID-19 infection status. In particular, if people were infected with COVID-19, they were worried about their physical conditions not meeting the requirements for blood donation (Table 4), indicating that COVID-19 infection had a psychological impact on people, which is consistent with the results of previous studies [31]. It also fits with the perception that people who have experienced infection, in general, perceive a higher risk than those who have not [32]. Compared to those in the nonpandemic stage of COVID-19 in Zhejiang province (Q Hu, unpublished data, March 2022), the 4 items with the highest degree of public worry during the COVID-19 pandemic period were blood donation may lead to infection with diseases, blood donation will cause temporary weakness, blood donation is harmful to health, and worries of family or friends regarding blood donation (Multimedia Appendix 1). The first 3 items were health-related concerns. This reflects that in the context of the COVID-19 outbreak, most of the public's attention was focused on COVID-19 and the perceived threat, which included the perceived susceptibility and the severity of infection. At that time, the public was highly sensitive to how the disease would affect their bodies and whether they would contract the infection, which is consistent with that reported in India, wherein the main reason that people did not donate blood (rate of 55%) was the fear of COVID-19 infection [33]. Other studies in the literature have shown that the higher the perceived threat, the more worry is triggered, which motivates people to adopt self-protective behaviors to control the danger [34-36]. In other words, the motivation to protect oneself from the disease takes over. In this study, during the peak of the COVID-19 pandemic, the number of blood donors decreased sharply. Except for those who were unable to donate blood, such as those infected, we speculate that this decrease was related to self-protective behaviors, that is, people may have wanted to take care and were more willing to avoid behaviors that might be associated with an increased risk of infection.

The second worry of the public, in terms of the overall situation of the specific worries of the public, was that blood donation will cause temporary weakness and that their physical health condition does not meet the blood donation requirements (Table 3). From the perspective of planned behavior theory, these 2 points fall under the category of perceived behavioral control as an influencing factor. Perceived behavioral control refers to obstacles that reflect the past experiences and expectations of the individual. The more resources and opportunities the individual thinks they have and the fewer obstacles they expect, the stronger is the perceived control of the behavior, that is, the degree of control (or mastery) the individual expects to feel when adopting a specific behavior [37]. The results of this study indicated that the public had less confidence in their ability to donate blood during the COVID-19 pandemic and expected that their ability to donate blood would decrease in the future. The COVID-19 pandemic is different from previous emergencies such as 9/11, in which people had high short-term enthusiasm for blood donation. However, the COVID-19 pandemic was different; it was a threat to the health of the whole population and their ability to donate blood, which was reflected in the blood donation behavior and the decline of the public's ability to donate blood and perceived behavior control.

The third worry of the public was that the blood donation site’s location and working hours were inconvenient. We speculated that this was related to the public health initiative to avoid gathering as much as possible owing to the rapid transmission of COVID-19. The distance from blood donation sites or inconvenient opening hours increased the risk of public travel by public transport, which could lead to higher infection rates.

The fourth worry of the public was that family or friends would be worried if they participated in blood donation, thereby reflecting the subjective norm level. Subjective norm refers to the social pressure to adopt a particular behavior, that is, the pressure from significant others or team members to believe that one should or should not perform a particular behavior. In the COVID-19 pandemic environment, as most people were at home at the time, the approval of blood donation by family and friends also became an important factor for respondents to consider. Not only did the public pay more attention to their own health but also their family and friends would influence their views on participation in blood donation and suggest postponing blood donation. These worries and suggestions could be pressurizing and contribute to reduction in blood donation.

This study also shows that worries regarding the harmful effects of blood donation on health during the COVID-19 pandemic were significantly higher than those during the non–COVID-19 pandemic period (Multimedia Appendix 1). These worries reflect the public's basic attitude toward blood donation, thereby creating a negative blood donation behavior. BTSs and the society should guide and correct this negative attitude. Therefore, the COVID-19 pandemic has reduced the public's perceived behavior control ability and subjective norms of blood donation to a certain extent and has had a negative impact on attitudes toward blood donation. However, family or friends would always be worried about blood donation, regardless of whether it was during the prepandemic period or the pandemic period. Worries regarding adverse reactions to blood donation, dissatisfaction with the previous blood donation experience, and anxiety about blood donation were more prevalent in the pre-epidemic period. This suggests that some worries about blood donation will persist, and BTSs should focus on addressing these long-standing worries from the public’s point of view.

### Factors Influencing Blood Donation Worries Regarding COVID-19

In addition to COVID-19 status, the factors influencing blood donation worries regarding COVID-19 included worries about the adverse reactions to blood donation, dissatisfaction with the blood donation experience, and worries of family or friends regarding blood donation (Table 4). However, the level of worries for the former 2 items was significantly lower than that during the non–COVID-19 pandemic period (Multimedia Appendix 1). We speculate that this may be because people's concerns were focused on physical health and how to avoid infection in the COVID-19 pandemic stage. At that time, there were still worries about adverse reactions and unsatisfactory experiences with blood donation, but these were not the main worries. However, if the respondent had been infected or if their current health condition made it impossible to participate in blood donation, worries about blood donation itself would appear. Thus, BTSs should focus on improving the blood collection technology and blood donation service, thereby reducing the occurrence of adverse reactions to the blood donated, improving the satisfaction with the blood donation experience, and improving their own service level. Concurrently, it is necessary to continue to follow up on publicizing voluntary blood donation in the society and targeting the families and friends of blood donors. Once worries about the infections have been resolved, the safety of the donation process, the experience, and the support of family and friends are the main concerns.

### Impact of the COVID-19 Pandemic on Blood Donation Intention

This study shows that male sex, older age, and more previous blood donations were associated with higher intention to donate blood, which were consistent with previous literature results [38,39]. In particular, previous blood donation experience was significantly correlated with blood donation intention (β=1.599), which verified that past behavior was the best predictor of future behavior [40-42]. This study also investigates the effect of blood donation worries regarding COVID-19 as a mediating factor for blood donation intention. The results indicated that respondents with blood donation worries regarding COVID-19 had a lower intention to donate blood compared to those who clearly stated that they did not have blood donation worries regarding COVID-19. This also confirms that the change in self-protection behavior is closely related to worry and the correlation strength is high [43]. In addition, one of the direct influencing factors of blood donation intention was the attitude that blood donation is harmful to health. The deeper this worry, the lower was the intention to donate blood. Negative attitudes toward blood donation can lead to a decrease in blood donation intention, which should be considered. Similar to that reported in previous studies [44,45], this study also verifies that anxiety about blood donation is a direct influencing factor of blood donation intention, and reducing blood donation anxiety can promote blood donation intention.

### Coping Strategy

Given the negative impact of the COVID-19 pandemic on the public's blood donation psychology and behavior, we suggest that BTSs should strengthen their publicity efforts, taking the recovery phase of COVID-19 as an important time point. This involves increasing the introduction of blood collection and supply processes as well as operating norms. BTSs should take efforts to reduce the public's concerns that blood donation may cause temporary physical weakness and infection, address the negative perceptions of blood donation, boost the public's confidence in their ability to donate blood, enhance their perceived behavioral control, alleviate concerns of family and friends to some extent, and improve subjective norms. In the medium and long term, whether the public's blood donation worries regarding COVID-19 will gradually decrease and whether the intention to donate blood will recover to previous levels still needs further observation.

### Limitations of This Study

The scale designed in this study has added factors such as COVID-19 and mainly discussed blood donation worries regarding COVID-19 and the influencing factors of blood donation intention. This scale is not exactly the same as the scale used in March 2022 (Q Hu, unpublished data) during the non–COVID-19 pandemic stage. The positive and negative expressions of sentences used in our scale may have caused a certain bias in the respondents' conscious choices. In addition, this study only included the Zhejiang province of China; therefore, the sample has geographical limitations and is not representative of other places.

### Conclusions

Due to the rapid transmission of the novel coronavirus and the prevalence of infection during the COVID-19 pandemic, the public's worries about health have increased, which, coupled with other factors, affect their intention to donate blood. Our study identifies the main worries of the public, such as fitness of their physical health condition to donate blood, the location and working hours of the blood donation sites, and the influence of family and friends, and analyzes the underlying reasons, which included a reduction in the public's perceived behavior control ability, a decline of subjective norms, and a negative impact on the blood donation attitude. In response to these issues, BTSs should take relevant measures to correct the negative attitude toward blood donation and improve awareness of the ability to donate blood. Timely and effective response to public worries and measures to improve blood supply would be of great significance in identifying the focus of voluntary nonremunerated blood donation in the future.

## Appendix

Multimedia Appendix 1 Comparison of the worry items during the COVID-19 pandemic and during the nonepidemic stage.

## Abbreviations

BTS: blood transfusion service

## Data Availability

The data sets generated during or analyzed during this study are available from the corresponding author on reasonable request.
