# Supplementary figures and images for: Association Between Concerns About COVID-19 Infection and Blood Donation Intention: Cross-Sectional Survey Study Through a Mobile Communication Platform
Source: J Med Internet Res. 2023 Nov 9;25:e46588. doi: 10.2196/46588 (PMC10667984; doi:10.2196/46588)

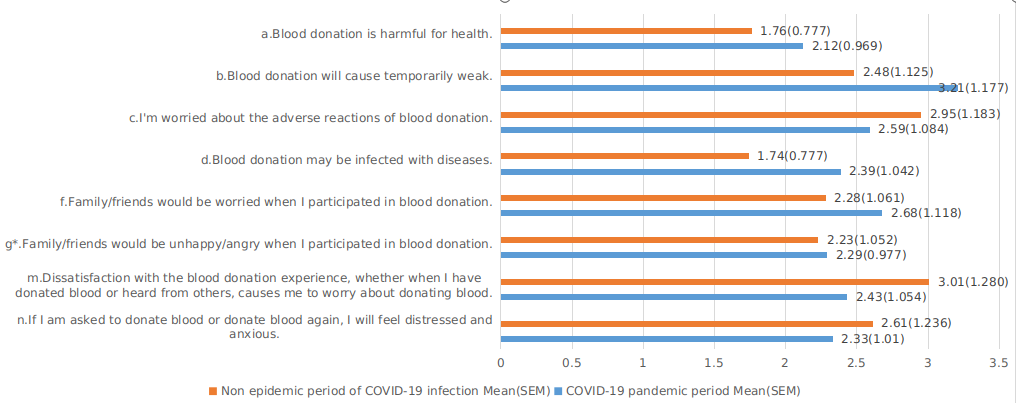

Supplement: Multimedia Appendix 1 [file jmir_v25i1e46588_app1.png]
